# An integrated genetic analysis of epileptogenic brain malformed lesions

**DOI:** 10.1186/s40478-023-01532-x

**Published:** 2023-03-02

**Authors:** Atsushi Fujita, Mitsuhiro Kato, Hidenori Sugano, Yasushi Iimura, Hiroharu Suzuki, Jun Tohyama, Masafumi Fukuda, Yosuke Ito, Shimpei Baba, Tohru Okanishi, Hideo Enoki, Ayataka Fujimoto, Akiyo Yamamoto, Kentaro Kawamura, Shinsuke Kato, Ryoko Honda, Tomonori Ono, Hideaki Shiraishi, Kiyoshi Egawa, Kentaro Shirai, Shinji Yamamoto, Itaru Hayakawa, Hisashi Kawawaki, Ken Saida, Naomi Tsuchida, Yuri Uchiyama, Kohei Hamanaka, Satoko Miyatake, Takeshi Mizuguchi, Mitsuko Nakashima, Hirotomo Saitsu, Noriko Miyake, Akiyoshi Kakita, Naomichi Matsumoto

**Affiliations:** 1 Department of Human Genetics, Yokohama City University Graduate School of Medicine, 3-9 Fukuura, Kanazawa-Ku, Yokohama, 236-0004 Japan; 2 Department of Pediatrics, Showa University School of Medicine, Tokyo, 142-8666 Japan; 3 Department of Neurosurgery, Epilepsy Center, Juntendo University, Tokyo, 113-8421 Japan; 4 Department of Child Neurology, National Hospital Organization Nishiniigata Chuo Hospital, Niigata, 950-2085 Japan; 5 Department of Functional Neurosurgery, Epilepsy Center, National Hospital Organization Nishiniigata Chuo Hospital, Niigata, 950-2085 Japan; 6 Department of Child Neurology, Comprehensive Epilepsy Center, Seirei Hamamatsu General Hospital, Hamamatsu, 430-8558 Japan; 7 Division of Child Neurology, Department of Brain and Neurosciences, Faculty of Medicine, Tottori University, Yonago, 683-8503 Japan; 8 Department of Pediatrics, Kawasaki Medical School, Kurashiki, 701-0192 Japan; 9 Comprehensive Epilepsy Center, Seirei Hamamatsu General Hospital, Hamamatsu, 430-8558 Japan; 10 Department of Pediatrics, Sapporo Medical University School of Medicine, Sapporo, 060-8543 Japan; 11 Department of Pediatrics, National Hospital Organization Nagasaki Medical Center, Omura, 856-8562 Japan; 12 Epilepsy Center, National Hospital Organization Nagasaki Medical Center, Omura, 856-8562 Japan; 13 Department of Pediatrics, Hokkaido University Graduate School of Medicine, Sapporo, 060-8638 Japan; 14 Department of Pediatrics, Tsuchiura Kyodo General Hospital, Tsuchiura, 300-0028 Japan; 15 Department of Neurosurgery, Tsuchiura Kyodo General Hospital, Tsuchiura, 300-0028 Japan; 16 Division of Neurology, National Center for Child Health and Development, Tokyo, 157-8535 Japan; 17 Department of Pediatric Neurology, Children’s Medical Center, Osaka City General Hospital, Osaka, 534-0021 Japan; 18 Department of Rare Disease Genomics, Yokohama City University Hospital, Yokohama, 236-0004 Japan; 19 Department of Clinical Genetics, Yokohama City University Hospital, Yokohama, 236-0004 Japan; 20 Department of Biochemistry, Hamamatsu University School of Medicine, Hamamatsu, 431-3192 Japan; 21 Department of Human Genetics, Research Institute, National Center for Global Health and Medicine, Tokyo, 162-8655 Japan; 22 Department of Pathology, Brain Research Institute, Niigata University, Niigata, 951-8585 Japan

**Keywords:** Focal cortical dysplasia, LEATs, mTOR, RAS/MAPK, Somatic variants

## Abstract

Focal cortical dysplasia is the most common malformation during cortical development, sometimes excised by epilepsy surgery and often caused by somatic variants of the mTOR pathway genes. In this study, we performed a genetic analysis of epileptogenic brain malformed lesions from 64 patients with focal cortical dysplasia, hemimegalencephy, brain tumors, or hippocampal sclerosis. Targeted sequencing, whole-exome sequencing, and single nucleotide polymorphism microarray detected four germline and 35 somatic variants, comprising three copy number variants and 36 single nucleotide variants and indels in 37 patients. One of the somatic variants in focal cortical dysplasia type IIB was an in-frame deletion in *MTOR*, in which only gain-of-function missense variants have been reported. In focal cortical dysplasia type I, somatic variants of *MAP2K1* and *PTPN11* involved in the RAS/MAPK pathway were detected. The in-frame deletions of *MTOR* and *MAP2K1* in this study resulted in the activation of the mTOR pathway in transiently transfected cells. In addition, the *PTPN11* missense variant tended to elongate activation of the mTOR or RAS/MAPK pathway, depending on culture conditions. We demonstrate that epileptogenic brain malformed lesions except for focal cortical dysplasia type II arose from somatic variants of diverse genes but were eventually linked to the mTOR pathway.

**Supplementary Information:**

The online version contains supplementary material available at 10.1186/s40478-023-01532-x.

## Introduction

Epileptogenic brain malformed lesions such as focal cortical dysplasia (FCD) and hemimegalencephaly (HME) are common causes of drug-resistant pediatric epilepsy [5, 17, 18, 24]. Epileptogenic lesions were resected in carefully selected patients suffering from these disorders, and approximately 60% of patients were seizure-free 1 year after the surgeries [5].

Genetic analyses for FCD type II and HME have focused on the PI3K-AKT3-mTOR pathway after identifying somatic variants of *MTOR, PIK3CA,* and *AKT3* as the gene aberrations [17, 18, 24]. Indeed, several genes belonging to this pathway have been associated with brain-malformed lesions [23]. In FCD type I or mild malformation of cortical development with oligodendroglial hyperplasia in epilepsy (MOGHE), somatic variants of *SLC35A2* encoding UDP‐galactose transporter have been reported [23, 36]. FCD type III comprises four subtypes based on cortical dyslamination with other epileptogenic lesions [23]. Genetic causes of FCD type IIIb with developmental brain tumor depend on tumor types. For instance, alterations in *BRAF* and *FGFR1* have been reported in low-grade epilepsy-associated tumors (LEATs) such as ganglioglioma and dysembryoplastic neuroepithelial tumors [32]. BRAF and FGFR1 regulate the RAS/MAPK pathway, and somatic variants of *KRAS* and *PTPN11* in this pathway have also been recently found in epileptogenic lesions [4, 21].

Somatic structural variations (SVs) have rarely been reported in FCD and HME. To our knowledge, chromosome 1q amplification was observed [4, 26], but no large somatic deletion has been reported. Loss-of-function variants causing FCD or HME have been identified in *TSC1, TSC2, DEPDC5*, *NPRL2*, and *NPRL3.* Germline deletions in these genes have been reported except in *NPRL2* [2, 29]. Therefore, somatic deletions in these genes are also expected in brain-malformed lesions, and somatic SVs have been detected in LEATs [21].

Here, we performed a genetic analysis of excised brain malformed lesions associated with intractable epilepsy such as FCD, HME, and brain tumors using targeted sequencing, whole-exome sequencing (WES), and single nucleotide polymorphism (SNP) array. In vitro cellular analyses were conducted to evaluate the status of the mTOR and RAS/MAPK pathways for novel aberrant genes and variants.

## Materials and methods

### Study subjects and samples

**Fig. 1:**
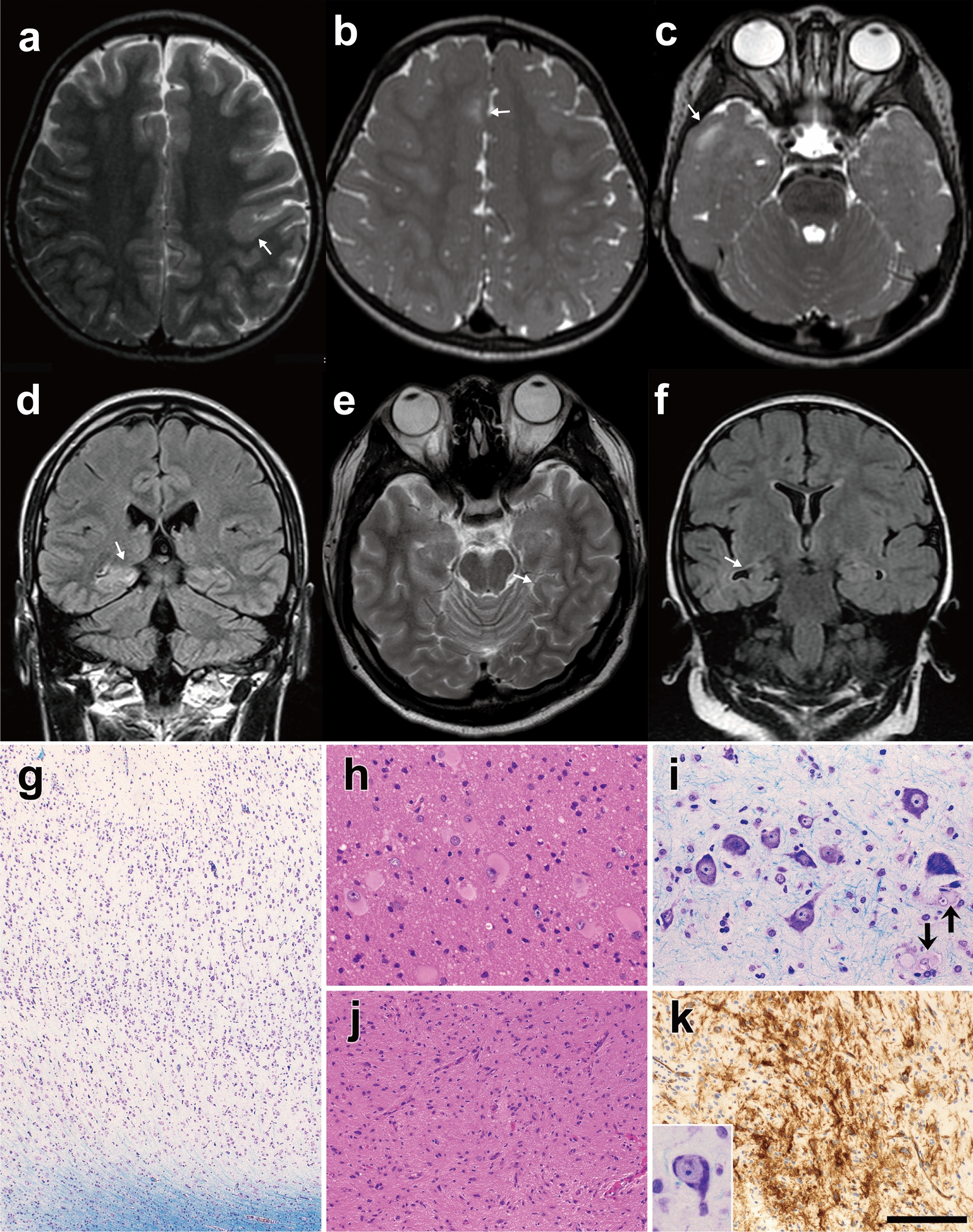
Brain MRI and histopathologic features of patients with somatic variants **a** Patient F08 at 3 years (FCD type IIB, an *MTOR* in-frame variant), **b, c** patient F61 at 2 years (FCD type IIB, germline and somatic variants of *TSC2*), **d** patient F48 at 23 years (FCD type IB, a *MAP2K1* in-frame deletion), **e** patient F70 at 21 years (ganglioglioma, deletion of entire chromosome 9), and **f** patient F30 at 2 years and 7 months (hippocampal sclerosis, 19p13.3p12 deletion). **a**, **b**, **c**, **e** are T2-weighted axial brain MRIs, and **d**, **f** are fluid-attenuated inversion recovery coronal MRIs. Brain MRI showing focal irregular gyri (arrows) with blurred junctions between the cortex and white matter (**a**) or hyperintensity of the subcortical white matter (**b**–**e**). **g** Patient F67 (FCD type I, a *PTPN11* missense variant). Cytoarchitectural abnormality of the cortex. **h** Patient F08 with FCD type IIB showing several balloon cells in the white matter. **i** Patient F61 with FCD type IIB exhibiting dysmorphic neurons and balloon cells (arrows) in the cortex. **j**, **k** Patient F70 with ganglioglioma. **j** Astrocytic cells showing a wavy, fascicular arrangement with mild hypercellularity. A dysmorphic ganglion cell (inset in k). **k** CD34-immunopositive cells with fine processes. **g**, **i** and inset in **k**: Klüver–Barrera stain. **h**, **j** hematoxylin and eosin stain. **k** immunostained and counterstained with hematoxylin. Scale bar = 350 μm for **g**, 90 μm for **h** and **i**, 180 μm for **j**, 50 μm for inset in **k**, and 140 μm for **k**

The study protocols were approved by the institutional review board of Yokohama City University School of Medicine. Written informed consent was obtained from all study participants. A total of 64 patients, including 18 previously reported patients without candidate variants (patients F02–F25) [24], participated in this study: FCD (*n* = 44), HME (*n* = 10), brain tumors (*n* = 4), hippocampal sclerosis (*n* = 1), and other malformations of cortical development (*n* = 5) (Fig. 1, Additional file 1: Figs. S1 and S2, and Table S1). Peripheral blood, saliva, frozen brain tissues, or formalin-fixed paraffin-embedded brain tissues were collected from patients. The brain lesions were surgically excised to alleviate intractable epilepsy. Genomic DNA of the peripheral blood leukocytes and FFPE brain tissues were extracted using QuickGene-610L (KURABO, Osaka, Japan) and QIAamp DNA FFPE Tissue Kit (Qiagen, Hilden, Germany), respectively. Genomic DNA samples were extracted from frozen brain tissues using the standard procedure involving proteinase K digestion, phenol–chloroform extraction, and ethanol precipitation.

### Deep sequencing using targeted capture

Targeted capture was conducted for DNA samples from brain tissues using the HaloPlex HS Target Enrichment System (Agilent Technologies, Santa Clara, CA, USA) and/or xGen Lockdown Probes with the xGen Prism DNA Library Prep Kit (Integrated DNA Technologies [IDT], Coralville, IA, USA). The data of HaloPlex HS were analyzed using the SureCall software (Agilent Technologies). xGen sequencing data were processed using the Picard, bwa, fgbio, and VarDict software according to the guidelines from IDT and were annotated using Annovar. The thresholds for variant allele frequency (VAF) in both analyses were set at 0.5%. The targeted captures covered 56–270 genes, depending on the capture kits used. These captures included genes associated with the mTOR and RAS/MAPK pathways and genes associated with diseases other than FCD because we used these targeted captures for diseases other than FCD. We filtered the variants based on the following conditions: exclusion of synonymous variants and other variants with a minor allele frequency (MAF) > 0.01 from 1000 Genomes Project, dbSNP138, or Human Genetic Variation Database in Haloplex; and exclusion of synonymous variants and variants with MAF > 0.01 from dbSNP138, NHLBI Exome Sequencing Project (ESP) 6500, The Exome Aggregation Consortium (ExAC), or Tohoku University Tohoku Medical Megabank Organization (ToMMo) 3.5 K in xGen. Then, the remaining variants were assessed using the prediction tools SIFT, Polyphen2, MutationTaster, and CADD, and the sequencing reads were manually checked using the Integrative Genomics Viewer (IGV). As for HaloPlex HS Target Enrichment, the mean depth of coverage was 1249 × (range, 334–6401×), and 96.8% (range, 86.3–99.3%) of the targeted regions was covered by at least 200 reads. For xGen Lockdown Probes, the mean depth of coverage was 1059× (range, 659–1567×), and 95.2% (range, 90.5–97.2%) of the coding region of the targeted Refseq genes was covered by at least 200 reads. Most cases were initially analyzed by targeted capture sequencing, and WES was applied if no candidate variants were found.

### WES

We performed WES using the SureSelect XT Human All Exon v5 or v6 kit (Agilent Technologies) or Twist Human Comprehensive Exome Panel (Twist Bioscience, San Francisco, CA, USA). DNA samples from only the brain or blood and brain samples were used. Germline variants were analyzed using GATK UnifiedGenotyper, and somatic variant calling was performed using Mutect2 and Varscan2. The threshold for VAF for somatic variants was set at 5%. In somatic analysis, we focused on common genes between unrelated samples or the genes in the list we made for targeted capture. The mean depth of coverage was 187 × (range, 55–300×) for brain tissues and 176 × (range, 76–288×) for blood or saliva.

### Copy number variant (CNV) analysis

To detect somatic CNVs, we analyzed WES data using the eXome-Hidden Markov Model (XHMM) [22, 34] and/or the SNP array with CytoScan HD array (Thermo Fisher Scientific, Waltham, MA, USA) according to the manufacturer’s instructions. CNVs were confirmed using the SNP array for patients F43 and F70 and droplet digital PCR (ddPCR) for patient F30. ddPCR was performed using the QX200 Droplet Digital PCR System and analyzed using QuantaSoft (both Bio-Rad Laboratories, Hercules, CA, USA). *CACNA1A* and *STK11* probes for the deleted region, *TSC2* probes as the non-deleted control, and *TERT* probes as an internal control for ddPCR were purchased from IDT. Three technical replicate measurements were repeated three times, meaning that a total of nine reactions were performed.

### Validation of somatic variants

We performed deep sequencing to validate the somatic candidate variants using the 150–300 bp long PCR amplicons. PCR and sequencing library preparation were performed with PrimeSTAR GXL DNA Polymerase (TaKaRa Bio, Shiga, Japan) and ThruPLEX DNA-Seq Kit (TaKaRa Bio). Libraries were sequenced on the Illumina platform, and data were analyzed as for WES but without PCR deduplication. The read count for VAF was manually inspected using IGV. The variants c.4376C > A, c.4379T > C, and c.6644C > A in *MTOR* were confirmed with ddPCR, as previously described [24]. For the blood sample of patient F68, we performed allele-specific PCR for the very-low-frequency somatic 5 bp deletion, in addition to deep sequencing. Germline variants were validated using Sanger sequencing.

### Plasmid construction

FLAG-HA-pcDNA3.1 vector and pcDNA3-Flag-mTOR-wt vector were purchased from Addgene (plasmid # 52535 and # 26603) [15, 35], and Halo-tagged cDNA clones of *MAP2K1*, *PTPN11*, and *GAB1* were obtained from Promega (Madison, WI, USA). Site-directed mutagenesis was performed using the Prime STAR GXL polymerase (TaKaRa Bio) to introduce the variants c.4339_4353del or c.4379T > C (as a positive control) in *MTOR*, c.173_187del in *MAP2K1*, or c.178G > C in *PTPN11* into each plasmid; each created mutant clone was confirmed using Sanger sequencing.

### Cell culture and immunoblotting analysis

HEK293T cells were maintained in Dulbecco's modified Eagle's medium (DMEM; FUJIFILM Wako Chemicals, Osaka, Japan) supplemented with 10% fetal bovine serum and 1% penicillin–streptomycin. To evaluate the activity of the mTOR and RAS/MAPK pathways, these cells were transfected with wild-type or mutant vector of *MTOR*, *MAP2K1*, or *PTPN11* with or without *GAB1* using Polyethylenimine (PEI) Max (Polysciences, Warrington, PA, USA)*.*

Twenty-four hours after transfection, the cell medium was replaced with serum-starved DMEM. After 24 h of incubation, the medium for the analysis of *MTOR* or *MAP2K1* was replaced with Dulbecco’s phosphate-buffered saline with MgCl_2_ and CaCl_2_ (Sigma-Aldrich, St. Louis, MO, USA) for 1 h [30]. In the cells transfected with the *PTPN11* vector with or without *GAB1*, epidermal growth factor (EGF; Sigma-Aldrich) stimulation was performed in serum-starved conditions for 5–120 min. Then, these cells were lysed using lysis buffer containing 1% Triton X-100, 20 mM Tris–HCl (pH 7.4), 150 mM NaCl, and 1 mM EDTA supplemented with cOmplete Protease Inhibitor Cocktail and PhosSTOP (both from Roche Diagnostics, Basel, Switzerland). These samples were incubated on ice for 30 min and centrifuged at 20,000×*g* for 20 min at 4 °C. Laemmli sample buffer (Sigma-Aldrich) was added to the supernatant, and the sample was boiled at 95 °C for 5 min.

An equal amount of protein was electrophoresed on 4–12% NuPAGE SDS–PAGE gels (Thermo Fisher Scientific) and transferred to Polyvinylidene difluoride membranes (ATTO, Tokyo, Japan). The total and phosphorylated S6 and ERK proteins were quantified using separate gels with the same amount of protein applied. The membranes were blocked using EzBlock Chemi (ATTO) for 30 min and incubated overnight with the following primary antibodies at 4 °C: anti-FLAG M2 (1:4000 dilution, A8592, Sigma-Aldrich), anti-HaloTag (1:1000 dilution, G921A, Promega), anti-S6 ribosomal protein (1:2000 dilution, #2217, Cell Signaling Technology, Danvers, MA, USA), anti-p-S6 ribosomal protein (Ser235/236; 1:4000 dilution, #4858, Cell Signaling Technology), anti-ERK1/2 (1:2000 dilution, #4695, Cell Signaling Technology), and anti-p-ERK1/2 (Thr202/Tyr204; 1:2000 dilution, #9101, Cell Signaling Technology) antibodies. Horseradish peroxidase (HRP)-conjugated anti-rabbit antibody (1:10,000 dilution, 111-035-003, Jackson ImmunoResearch Laboratories, West Grove, PA, USA) or HRP-conjugated anti-mouse antibody (1:10,000 dilution, 115-035-003, Jackson ImmunoResearch Laboratories) was used as the secondary antibody and was incubated with the membranes for 2 h at room temperature. The membranes were developed using the SuperSignal West Dura Extended Duration Substrate (Thermo Fisher Scientific), and the bands were visualized using a ChemiDoc Touch imaging system (Bio-Rad Laboratories). The data were analyzed using the Image Lab software (Bio-Rad Laboratories). The experiments were performed in triplicate or quadruplicate.

### Statistical analysis

All statistical analyses were performed using GraphPad Prism 9 (GraphPad Prism Software, San Diego, CA, USA). Statistical differences were analyzed by one-way ANOVA followed by Dunnett's post-hoc test for immunoblotting analysis of MTOR and ddPCR, two-tailed paired t-test for immunoblotting of MAP2K1, and two-way repeated measurement ANOVA with Sidak’s multiple comparison test for immunoblotting of PTPN11. The level of significance was set at *P* < 0.05.

## Results

### Summary of the genetic analysis

We identified 36 single nucleotide variants (SNVs)/indels and three CNVs in 37 of the 64 individuals, which included four germline and 35 somatic variants (Tables 1, 2 and Additional file 1: Tables S2 and S3). Somatic variants of the genes involved in the PI3K-AKT3-mTOR pathway were identified in FCD type II and HME. In contrast, somatic variants of the genes involved in the PI3K-AKT3-mTOR and RAS/MAPK pathways and of *SLC35A2* were identified in the other types of epileptogenic lesions.

**Table 1:** Somatic and germline variants of FCD type II in this study

ID	Gene	Variant	Method		VAF (%) in validation		Germline/somatic	Reported/novel variant
			Detection	Validation	Blood	Brain		
*FCD type IIA*								
F53	*DEPDC5*	c.982C > T p.(Arg328*)	TS	Sanger	NA	NA	Germline	Reported
F72	*PIK3CA*	c.1035T > A p.(Asn345Lys)	TS	DS	NA	12.8–16.8	Somatic	Reported
*FCD type IIB*								
F04	*TUBB3*	c.520A > G p.(Lys174Glu)	WES	Sanger	NA	NA	Germline	Novel
F08	*MTOR*	c.4339_4353del p.(Ala1447_Glu1451del)	WES	DS	0	1.8	Somatic	Novel
F36	*TSC2*	c.2492C > T p.(Thr831Met)	TS	DS	0.1^a^	1.4	Somatic	Novel
F38	*NPRL3*	c.629 + 2_3insT	TS	DS	32.5	32.9	Somatic	Novel
F40	*MTOR*	c.4447T > C p.(Cys1483Arg)	TS	DS	0	0.6	Somatic	Reported
F41	*TSC2*	c.5227C > T p.(Arg1743Trp)	TS	DS	0	1	Somatic	Novel
F43	*DEPDC5*	22q11.23q13.33del and 17q12q25.3dup	WES	CMA	0	20–25	Somatic	Novel
F43	*DEPDC5*	c.856C > T p.(Arg286*)	TS	DS	0.1^a^	1.8	Somatic	Reported
F44	*TSC2*	c.1513C > T p.(Arg505*)	TS	DS	0	1.6	Somatic	Novel
F46	*MTOR*	c.4376C > A p.(Ala1459Asp)	TS	ddPCR	0	2.56	Somatic	Reported
F49	*TSC2*	c.5228G > A p.(Arg1743Gln)	TS	DS	0.1^a^	1.3	Somatic	Reported
F50	*MTOR*	c.5930C > A p.(Thr1977Lys)	TS	DS	0	2.6	Somatic	Reported
F54	*MTOR*	c.4379T > C p.(Leu1460Pro)	TS	ddPCR	0	1.84	Somatic	Reported
F57	*MTOR*	c.6644C > A p.(Ser2215Tyr)	TS	ddPCR	0	3.18	Somatic	Reported
F61	*TSC2*	c.4375C > T p.(Arg1459*)	TS	DS	0	0.6	Somatic	Novel
F61	*TSC2*	c.4960G > A p.(Gly1654Ser)	TS	Sanger	NA	NA	Germline	Novel
F64	*MTOR*	c.4448G > A p.(Cys1483Tyr)	TS	DS	NA	4	Somatic	Reported
F68	*TSC2*	c.1418_1422del p.(Leu473Hisfs*7)	TS	DS/ASP	0.03–0.4	3.6	Somatic	Novel

*ASP* allele-specific PCR, *CMA* chromosomal microarray, *ddPCR* droplet digital PCR, *DS* amplicon deep sequencing, *NA* not available, *TS* targeted sequencing, *VAF* variant allele frequency, *WES* whole-exome sequencing

^a^These data were considered false positive due to similar error rates in the short-read sequencer (0.1%) (Additional file 1: Tables S2 and S3). Refseq accession number: *DEPDC5* (NM_001242896), *MTOR* (NM_004958), *NPRL3* (NM_001077350), *PIK3CA* (NM_006218), *TSC2* (NM_000548), and *TUBB3* (NM_006086)

**Table 2:** Somatic and germline variants in FCD type I and III, HME, and the other pathological types in this study

ID	Gene	Variant	Method		VAF (%) in validation		Germline/somatic	Reported/novel variant
			Detection	Validation	Blood	Brain		
*FCD type I*								
F26	*AKT3*	c.49G > A p.(Glu17Lys)	WES	DS	0	4.9	Somatic	Reported
F32	*BRAF*	c.1799T > A p.(Val600Glu)	TS	DS	0.3^a^	7.5	Somatic	Reported
F45	*SLC35A2*	c.844G > A p.(Gly282Arg)	WES	DS	0.1^a^	5.4	Somatic	Novel
F48	*MAP2K1*	c.173_187del p.(Gln58_Glu62del)	TS	DS	0	0.8	Somatic	Novel
F67	*PTPN11*	c.178G > C p.(Gly60Arg)	WES	DS	0.1^a^	2.3	Somatic	Novel
*Malformation of cortical development*								
F29	*MTOR*	c.4379T > C p.(Leu1460Pro)	WES	ddPCR	0	2.3	Somatic	Reported
*FCD type IIIb (Ganglioglioma and FCD type IA)*								
F39	*BRAF*	c.1799T > A p.(Val600Glu)	TS	DS	0	0, 29.1	Somatic	Reported
*Ganglioglioma*								
F70	*TSC1*	chr 9 del	WES	CMA	0	15–20	Somatic	Reported
*Hippocampal sclerosis*								
F30	–	19p13.3p12 del	CMA	ddPCR	0	20	Somatic	Novel
*No remarkable change (insufficient sample volume)*								
F63	*NPRL3*	c.1270C > T p.(Arg424*)	TS	Sanger	NA	NA	Germline	Reported
*Hemimegalencephaly*								
M28	*PIK3CA*	c.1624G > A p.(Glu542Lys)	TS	DS	0.2^a^	21.7, 34.9	Somatic	Reported
M29	*PIK3CA*	c.1624G > A p.(Glu542Lys)	TS	DS	0	27.2	Somatic	Reported
M30	*MTOR*	c.6644C > T p.(Ser2215Phe)	TS	ddPCR	0.016^a^	16.28	Somatic	Reported
M44	*MTOR*	c.4348T > G p.(Tyr1450Asp)	TS	DS	NA	4.2–6.2	Somatic	Reported
M52	*PIK3CA*	c.1633G > A p.(Glu545Lys)	TS	DS	NA	11.3	Somatic	Reported
M53	*PIK3CA*	c.3140A > G p.(His1047Arg)	TS	DS	0	16.8, 22.3 (brain) 15.2 (lipoma)	Somatic	Reported
M54	*AKT3*	c.49G > A p.(Glu17Lys)	TS	DS	0	1.1	Somatic	Reported
M55	*PIK3CA*	c.1624G > A p.(Glu542Lys)	TS	DS	0	25.4	Somatic	Reported
M56	*PIK3CA*	c.1633G > A p.(Glu545Lys)	Sanger	DS	0	7.1–14.6 (brain), 14.3 (skin)	Somatic	Reported

*CMA* chromosomal microarray, *ddPCR* droplet digital PCR, *DS* amplicon deep sequencing, *TS* targeted sequencing, *VAF* variant allele frequency, *WES* whole-exome sequencing

^a^These data were considered false positive because VAFs in the blood were smaller in controls as per ddPCR or similar to the error rate for the short-read sequencer (0.1%) (Additional file 1: Tables S2 and S3). Refseq accession number: *AKT3* (NM_005465), *BRAF* (NM_004333), *MAP2K1* (NM_002755), *MTOR* (NM_004958), *PIK3CA* (NM_006218), *PTPN11* (NM_002834), *SLC35A2* (NM_001042498)

In HME of this study, VAF showed a wide range of 1–35% depending on individuals and tissues, and the somatic variant was not detected in blood even when the brain VAF was high. In FCD, VAF was less than 4% in type IIB and less than 10% in type I. These data indicate that targeted deep sequencing is certainly suitable for detecting such low-prevalent somatic variants in FCD.

### Somatic variants in brain lesions and functional studies

**Fig. 2:**
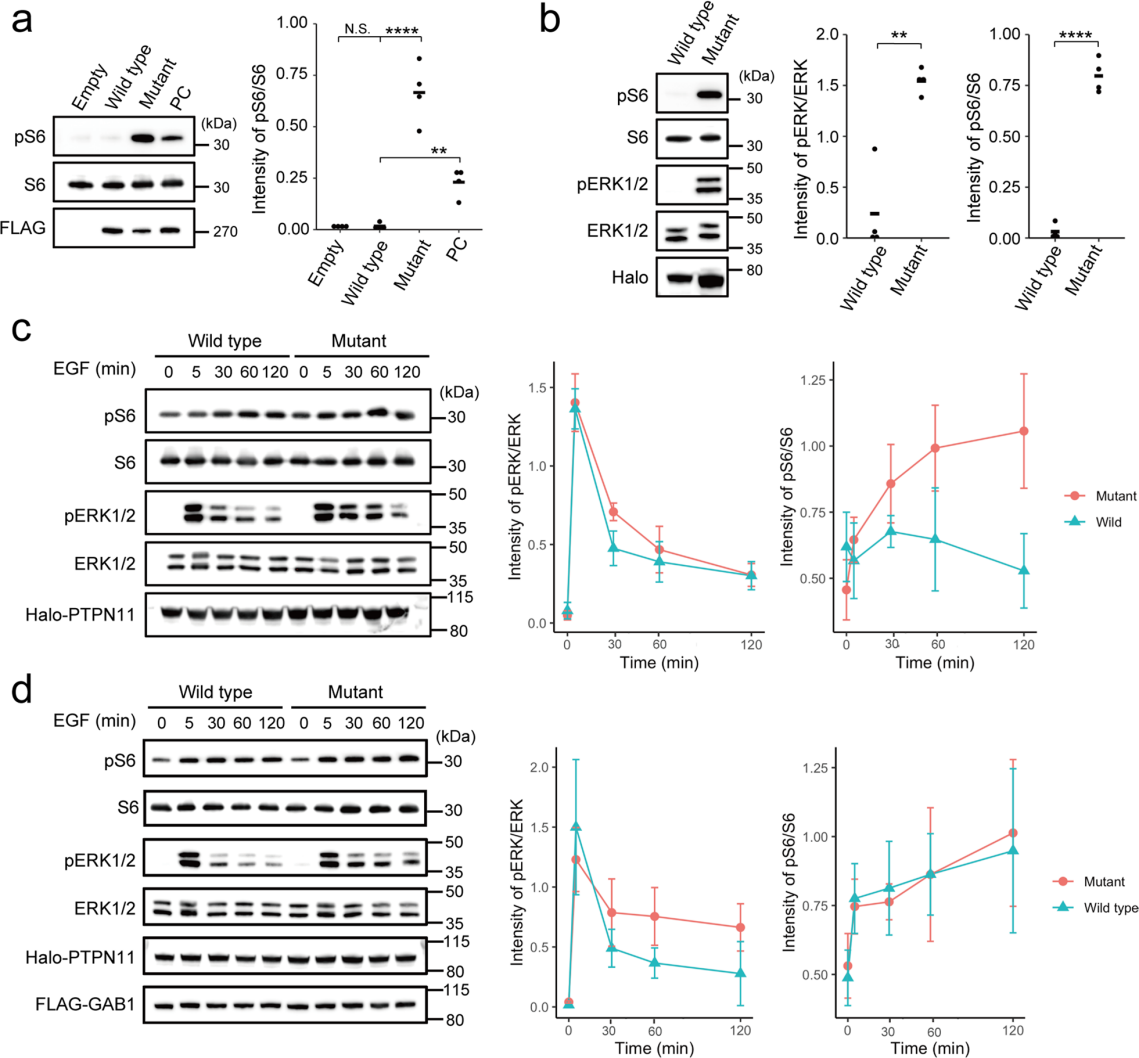
Immunoblotting analysis of HEK293T cells transfected with mutant *MTOR, MAP2K1*, and *PTPN11* **a** An in-frame (15-bp) *MTOR* deletion, c.4339_4353del p.(Ala1447_Glu1451del), detected in our study resulted in significantly higher phospho-S6 expression compared to wild-type (One-way ANOVA followed by Dunnett's post-hoc test; Empty: *P* = 0.99; Mutant: *P* =  < 0.0001; PC: *P* = 0.0079). Positive control (PC), c.4379T > C p.(Leu1460Pro). **b** The *MAP2K1* variant, c.173_187del p.(Gln58_Glu62del), resulted in significantly higher phospho-S6 and phospho-ERK levels compared to the wild-type (Two-tailed paired t-test; pERK/ERK: *P* = 0.0048; pS6/S6: *P* ≤ 0.0001). **c**, **d** Time course of relative expression of phospho-ERK and phospho-S6 under stimulation with EGF in cells with *PTPN11* transfection (**c**) and co-transfection with *PTPN11* and *GAB1* (**d**). Two-way repeated measurement ANOVA in **c**; pERK/ERK: *P* = 0.35; pS6/S6: *P* = 0.0028. Sidak’s post hoc test in pS6/S6 of c; 0 min: *P* = 0.63; 5 min: *P* = 0.96; 30 min: *P* = 0.58; 60 min: *P* = 0.34; 120 min: *P* = 0.14. Two-way repeated measurement ANOVA in d; pERK/ERK: *P* = 0.38; pS6/S6: *P* = 0.96. Bars in **a**, **b** represent the mean, and those in **c**, **d** represent SD. **P < 0.01; ****P < 0.0001. *N.S.* not significant

An *MTOR* in-frame deletion [c.4339_4353del p.(Ala1447_Glu1451del)] was confirmed in patient F08, which Mutect2 only detected, but not by the Varscan2 software, in addition to the missense variants in nine patients. This deletion was located at the FAT domain, the mutational hot spot for FCD, and *in-silico* analysis predicted this mutation to be deleterious (Additional file 1: Table S4). To our knowledge, an in-frame deletion of *MTOR* has not been reported. Therefore, we checked the effect of this variant on the activity of the mTOR pathway using transiently transfected HEK293T cells, and the phospho-S6 levels in the mutant-transfected cells were significantly increased compared to the wild-type, indicating the variant activated mTOR signaling (Fig. 2a).

An in-frame variant [c.173_187del p.(Gln58_Glu62del)] in *MAP2K1* and a missense variant [c.178G > C p.(Gly60Arg)] in *PTPN11* were identified in patients F48 and F67 with FCD type I, respectively. Mutations in these two genes have not been reported in FCD type I. MAP2K1 and PTPN11 are essential components of the RAS/MAPK pathway, which partially activates the mTOR pathway by phosphorylating ERK in the RAS/MAPK pathway and suppressing the TSC2 function [6, 29]. Thus, we checked the activation of the mTOR and RAS/MAPK pathway as described above for *MTOR.* The in-frame variant of *MAP2K1* activated both pathways (Fig. 2b), whereas the *PTPN11* missense variant led to no activity change in either of the two pathways compared to the wild-type (data not shown).

Next, we examined these two pathways by stimulation with EGF for the *PTPN11* variant. It led to no significant change in phospho-ERK and phospho-S6 levels between the wild-type and mutant. However, in the mutant, the phospho-ERK levels were slightly more maintained 60 min after stimulation compared to that in the wild-type, and phospho-S6 levels tended to be more maintained at least until 120 min (Fig. 2c). To more clearly recognize phospho-ERK, *PTPN11* and *GAB1* (a major binding partner of PTPN11) were co-transfected together with EGF treatment. The effect of the *PTPN11* variant is tissue- or cell-specific [1], but these experimental conditions previously showed that the gain-of-function variant in *PTPN11* prolonged phospho-ERK [11]. In this experiment, phospho-ERK in the mutant was sustained but with no significant difference from that in the wild-type (Fig. 2d). However, phospho-S6 did not show a similar pattern to that in the experiment without *GAB1* overexpression (Fig. 2c).

### Somatic SVs

In patients F43 and F70, large CNVs occurred in known aberrant genes for FCD, i.e., *DEPDC5* and *TSC1*, respectively. These were initially suspected based on WES data and confirmed using the SNP array (Additional file 1: Figs. S3a–c and Table S5). Interestingly, the patient F43 showed 43-Mb duplication at 17q12-q25.3 in addition to 26-Mb deletion at 22q11.23-q13.33 involving *DEPDC5* possibly through an unbalanced somatic translocation together with a somatic SNV [c.856C > T p.(Arg286*)] in *DEPDC5*. This led to a 2-hit alteration of *DEPDC5*. Patient F70 showed a deletion in the entire chromosome 9, which affected *TSC1*. Patient F30 with hippocampal sclerosis had a deletion at 19p13.3-p12 and copy-neutral loss of heterozygosity at 5q34-qter recognized by the SNP array despite the low-quality data. The deletion was validated using ddPCR (Additional file 1: Figs. S3d and S4, Table S5).

### Germline and somatic variants not specific to brain tissues

The four germline variants found in this study were de novo (*TUBB3*), paternally inherited (*DEPDC5*), or of unknown inheritance (*TSC2* and *NPRL3*). DEPDC5, NPRL2, and NPRL3 form the GATOR complex, and pathogenic variants of the genes encoding them exhibit incomplete penetrance [2]. Patient F61 showed a somatic nonsense variant [c.4375C > T p.(Arg1459*)] in *TSC2,* along with a *TSC2* germline variant [c.4960G > A p.(Gly1654Ser)] of unknown origin, which has been predicted as disease-causing (Additional file 1: Table S4), implying a two-hit event in *TSC2*; however, further studies are required to confirm if they reside on different alleles. Regarding somatic variants in both the brain tissues and blood, the *NPRL3* splicing variant (c.629 + 2_3insT) in the lymphoblastoid cells of patient F38 caused abnormal splicing associated with nonsense-mediated mRNA decay (Additional file 1: Fig. S5), and patient F68 had a very low-frequency somatic variant of *TSC2* in the blood and brain lesion (Additional file 1: Fig. S6).

## Discussion

Somatic variants of PI3K-AKT3-mTOR pathway genes were consistently found in epileptogenic lesions of our patients with FCD type II and HME. Additionally, somatic variants of other genes involved in the RAS/MAPK pathway and of *SLC35A2* were detected in FCD type I and LEATs. Functional analysis of somatic variants in FCD type I indicated RAS/MAPK and mTOR pathway activation, indicating common etiology among epileptogenic brain malformed lesions.

The current and previous studies detected somatic *PTPN11* variants in FCD type I and in FCD type IIIa, IIId, LEAT, and hippocampal sclerosis, respectively [4, 21, 28]. We also reported somatic variants of *PTPN11* in hypothalamic hamartoma [12] with epileptogenicity originating during fetal development, such as FCD.

Germline variants of *PTPN11* cause Noonan syndrome 1 (OMIM# 163950) and LEOPARD syndrome 1 (OMIM# 151100). *PTPN11* encodes SHP2, which contains two tandem Src homology-2 (N-SH2 and C-SH2) domains and a protein tyrosine phosphatase (PTP) domain. The catalytic PTP function is autoinhibited via a blocking interaction between the N-SH2 and PTP domains [14, 33]. Most germline *PTPN11* variants in patients with Noonan syndrome are located at the interaction site, disrupting autoinhibition with gain-of-function mutations [11, 33]. p.(Gly60Arg) identified in this study is located at the N-SH2 domain resembling variants in Noonan syndrome. The same germline variant has been reported in two patients with RASopathy or no informative phenotype and interpreted as pathogenic or likely pathogenic (ClinVar Accession: VCV000372590.4). An identical somatic variant has been reported in juvenile myelomonocytic leukemia, but no functional studies were conducted [19].

Co-transfection of the mutant *PTPN11* with *GAB1* could maintain the activation of ERK under EGF stimulation, suggesting prolonged activation of the RAS/MAPK pathway due to the *PTPN11* mutant. However, phospho-S6 levels were increased in both mutant and wild-type, possible due to *GAB1* overexpression. In a normal bladder cell line, the mTOR pathway is activated by *GAB1* overexpression, probably through the PI3K/AKT pathway [8]. Furthermore, HEK293 cells overexpressing *GAB1* with the Noonan syndrome-causing *PTPN11* variant or the wild-type did not differ in the PI3K activation levels [10]. Therefore, in our experiment, S6 might be phosphorylated through the PI3K/AKT pathway in both the wild-type and p.(Gly60Arg) variant. We only evaluated the effect of the RAS/MAPK pathway in this context.

In this study, a somatic in-frame deletion, p.(Gln58_Glu62del), in MAP2K1 was identified in FCD type I. Germline variants of *MAP2K1* have been reported in patients with RASopathy, such as cardio-facio-cutaneous syndrome [27]. The other somatic in-frame deletions involving the same amino acid residues (p.Gln58_Glu62) have been reported in multinodular and vacuolating neuronal tumors, a low-grade neuronal neoplasm of the cerebral hemispheres associated with adult-onset seizures [25]. Our patient with a somatic *MAP2K1* variant developed epilepsy at the age of 4 years and underwent surgery at 23. The age of onset and pathological difference of the affected tissues may be influenced by when and where the somatic variant occurred. Identical somatic variants have also been reported in Langerhans cell histiocytosis, which is a myeloproliferative disorder, and this variant upregulated the RAS/MAPK pathway [7]. We found that the mTOR and RAS/MAPK pathways were both activated in cells with the *MAP2K1* mutant. As shown by the transfection experiments of *PTPN11* and *MAP2K*, while the solitary activation of the mTOR pathway causes FCD type II and HME, the abnormal activation of both the mTOR and RAS/MAPK pathways during brain development may lead to other types of epileptogenic lesions such as LEATs or FCD types I and III.

We identified large SVs in three patients in this cohort. In patients F43 with FCD type IIB and F70 with ganglioglioma, *DEPDC5* and *TSC1* were deleted, respectively, together with many other genes through somatic chromosomal deletions. Similar somatic large CNVs have recently been reported in LEATs [21]. Data from our cohort and another study suggest that even if the mTOR pathway genes are deleted, such CNVs can cause LEATs and not FCD, possibly due to the influence of other ablated genes within a large CNV. The remaining somatic SV in patient F30 with hippocampal sclerosis is a 23.5-Mb deletion at 19p13.3-p12 and copy-neutral loss of heterozygosity at 5q34-qter. However, these somatic SVs have never been reported in epileptogenic tissues. Furthermore, brain basal ganglia or frontal cortex in two normal individuals have somatic chromosome 19p deletion with 19–27% of VAF detected by RNA sequencing in GTEx samples [13]. Therefore, the effect in F30 is inconclusive.

Genetic data of epileptogenic brain malformed lesions could provide helpful information regarding molecular targeted therapy and diagnosis through neuroimaging and histopathology [23, 32]. Somatic variant detection in epileptogenic lesions was attempted using cell-free DNA in cerebrospinal fluid, and variants were indeed found in some cases [16]. Because FCD harbored somatic variants with low VAF in affected tissues and could be even lower in cell-free DNA, it is essential to develop much more sensitive methods to detect very low VAF somatic variants in cell-free DNA accurately.

Somatic variants of FCD are enriched in neuronal cells compared to non-neuronal cells as determined by analysis using laser capture microdissection, and dysmorphic neurons and balloon cells density in bulk tissue is an important implication in detecting somatic variants [3, 9]. Spatially resolved DNA analysis, which has recently been applied to cancer tissues to detect site-specific small variants or genome-wide CNVs, allows unbiased analysis including surrounding normal cells [20, 31, 37], and somatic variant analysis with spatial information may provide significant information for understanding the development of FCD.

In summary, somatic variants of the mTOR pathway genes were confirmed in FCD type II and HME, as previously reported. Furthermore, somatic variants of the RAS/MAPK pathway genes and SVs of genes such as the mTOR pathway genes may also activate the mTOR pathway, similar to FCD type II and HME.

## Supplementary Information

**Additional file 1**:** Fig. S1**. Brain MRI studies of patients harboring pathogenic variants in this study; **Fig. S2**. Brain MRI studies of patients with hemimegalencephaly harboring somatic variants; **Fig. S3**. Results of the SNP array in three patients in this study; **Fig. S4**. Confirmation of somatic deletion of chromosome 19 in affected brain tissue from patient F30 using ddPCR; **Fig. S5**. cDNA analysis of a somatic splicing variant of *NPRL3* in lymphoblastoid cells from patient F38; **Fig. S6**. Allele-specific PCR for *TSC2* somatic variant in the peripheral blood leukocytes from patient F68; **Table S1**. Pathological types and genetic analyses of the 64 patients enrolled in this study; **Table S2**. Clinical summary and genetic variants in patients with FCD or other pathological types in this study; **Table S3**. Clinical summary and genetic variants in patients with hemimegalencephaly in this study; **Table S4**. In-silico predictions of novel missense and in-frame variants identified in this study; **Table S5**. Somatic structural variations identified in this study.

## Data Availability

The data supporting the findings of this study are available from the corresponding author upon request.
