# Supplementary material for: An integrated genetic analysis of epileptogenic brain malformed lesions
Source: Acta Neuropathol Commun. 2023 Mar 2;11:33. doi: 10.1186/s40478-023-01532-x (PMC9983246; doi:10.1186/s40478-023-01532-x)
Supplement: Supplementary file 1 — Additional file 1: Fig. S1. Brain MRI studies of patients harboring pathogenic variants in this study; Fig. S2. Brain MRI studies of patients with hemimegalencephaly harboring somatic variants; Fig. S3. Results of the SNP array in three patients in this study; Fig. S4. Confirmation of somatic deletion of chromosome 19 in affected brain tissue from patient F30 using ddPCR; Fig. S5. cDNA analysis of a somatic splicing variant of NPRL3 in lymphoblastoid cells from patient F38; Fig. S6. Allele-specific PCR for TSC2 somatic variant in the peripheral blood leukocytes from patient F68; Table S1. Pathological types and genetic analyses of the 64 patients enrolled in this study; Table S2. Clinical summary and genetic variants in patients with FCD or other pathological types in this study; Table S3. Clinical summary and genetic variants in patients with hemimegalencephaly in this study; Table S4. In-silico predictions of novel missense and in-frame variants identified in this study; Table S5. Somatic structural variations identified in this study. [file 40478_2023_1532_MOESM1_ESM.pdf]

Supplementary information

An integrated genetic analysis of epileptogenic brain malformed lesions

Atsushi Fujita,[1] Mitsuhiro Kato,[2] Hidenori Sugano,[3] Yasushi Iimura,[3] Hiroharu Suzuki,[3] Jun Tohyama,[4] Masafumi Fukuda,[5] Yosuke Ito,[5] Shimpei Baba,[6] Tohru Okanishi,[7] Hideo Enoki,[8] Ayataka Fujimoto,[9] Akiyo Yamamoto,[10] Kentaro Kawamura,[10] Shinsuke Kato,[10] Ryoko Honda,[11] Tomonori Ono,[12] Hideaki Shiraishi,[13] Kiyoshi Egawa,[13] Kentaro Shirai,[14] Shinji Yamamoto,[15] Itaru Hayakawa,[16] Hisashi Kawawaki,[17] Ken Saida,[1] Naomi Tsuchida,[1,18] Yuri Uchiyama,[1,18] Kohei Hamanaka,[1] Satoko Miyatake,[1,19] Takeshi Mizuguchi,[1] Mitsuko Nakashima,[1,20] Hirotomo Saitsu,[1,20] Noriko Miyake,[1,21] Akiyoshi Kakita,[22] Naomichi Matsumoto[1]*

Correspondence to: Naomichi Matsumoto

Department of Human Genetics, Yokohama City University Graduate School of Medicine

3-9 Fukuura, Kanazawa-ku, Yokohama 236-0004, Japan

E-mail naomat@yokohama-cu.ac.jp

Tel +81-45-787-2606

Fax +81-45-786-5219

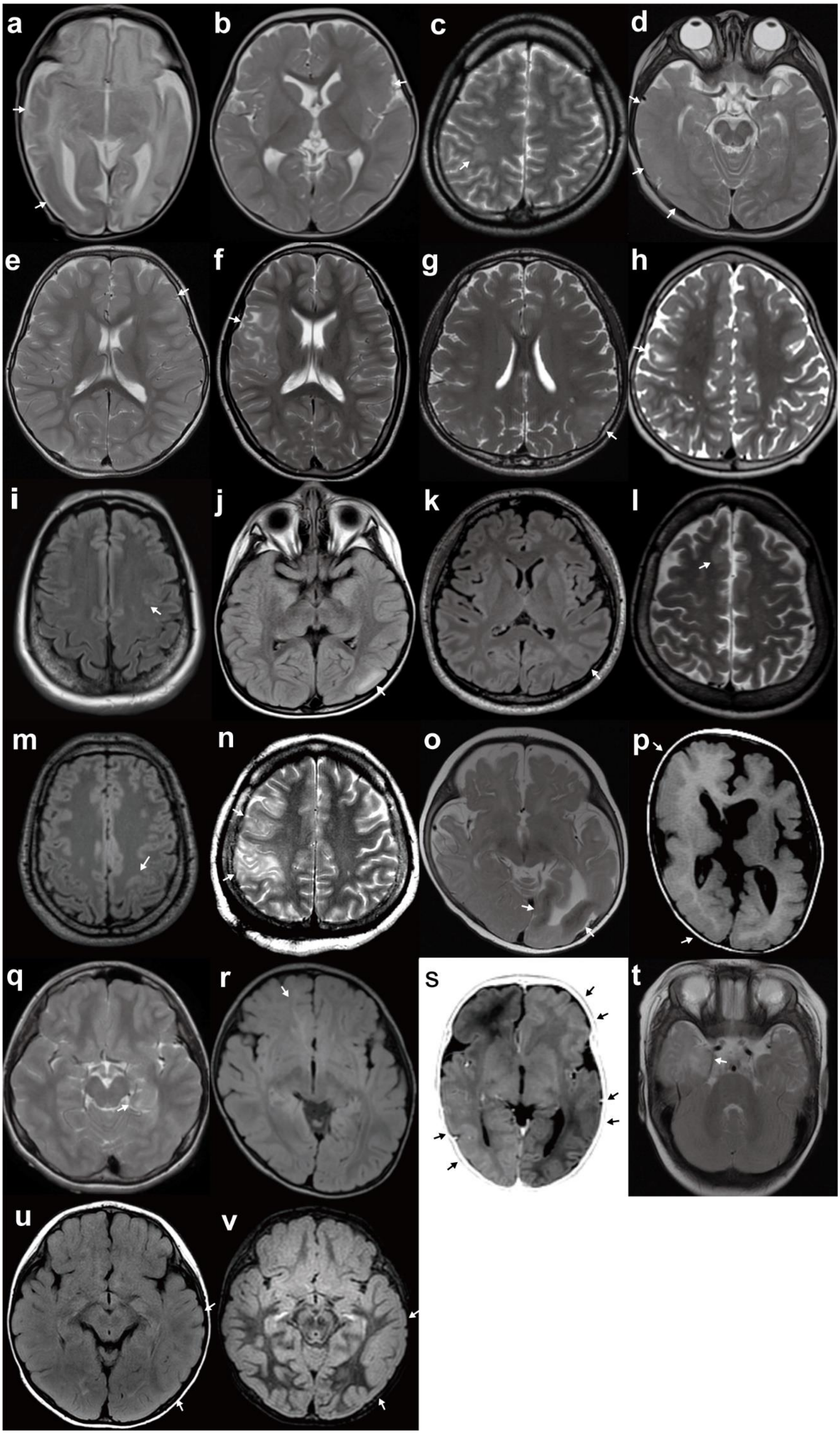

a
b
c
d
e
f
g
h
i
j
k
l
m
n
o
p
q
r
s
t
u
v

Fig. S1 Brain MRI studies of patients harboring pathogenic variants in this study (a) Patient F53 at the age of 20 days, **(b)** patient F72 at 15 months, **(c)** patient F04 at 27 years, **(d)** patient F36 at 16 months, **(e)** patient F38 at 2 years, **(f)** patient F40 at 13 years, **(g)** patient F41 at 13 years, **(h)** patient F44 at 6 years, **(i)** patient F46 at 39 years, **(j)** patient F49 at 4 years, **(k)** patient F50 at 25 years, **(l)** patient F54 at 53 years, **(m)** patient F57 at 15 years, **(n)** patient F64 at 56 years, **(o)** patient F68 at 9 months, **(p)** patient F26 at 1 year, **(q)** patient F32 at 12 years, **(r)** patient F45 at 1 year, **(s)** patient F29 at 3 days, **(t)** patient F39 at 5 months, and **(u, v)** patient F63 at 16 months. T2-weighted brain MRI (a-h, j-l, n, o, q, t, and u), fluid-attenuated inversion recovery (i, m, r), T1-weighted MRI (p), T2-reversed MRI (s), and double inversion recovery MRI (v) are shown. Brain MRI showed bilateral (p and s) or focal (others) irregular gyri (arrows) with blurred junctions between the cortex and white matter (b, d, e, and g) or hyperintensity of the subcortical white matter (c, f, h, i, j, k, m, n, q, r, and t).

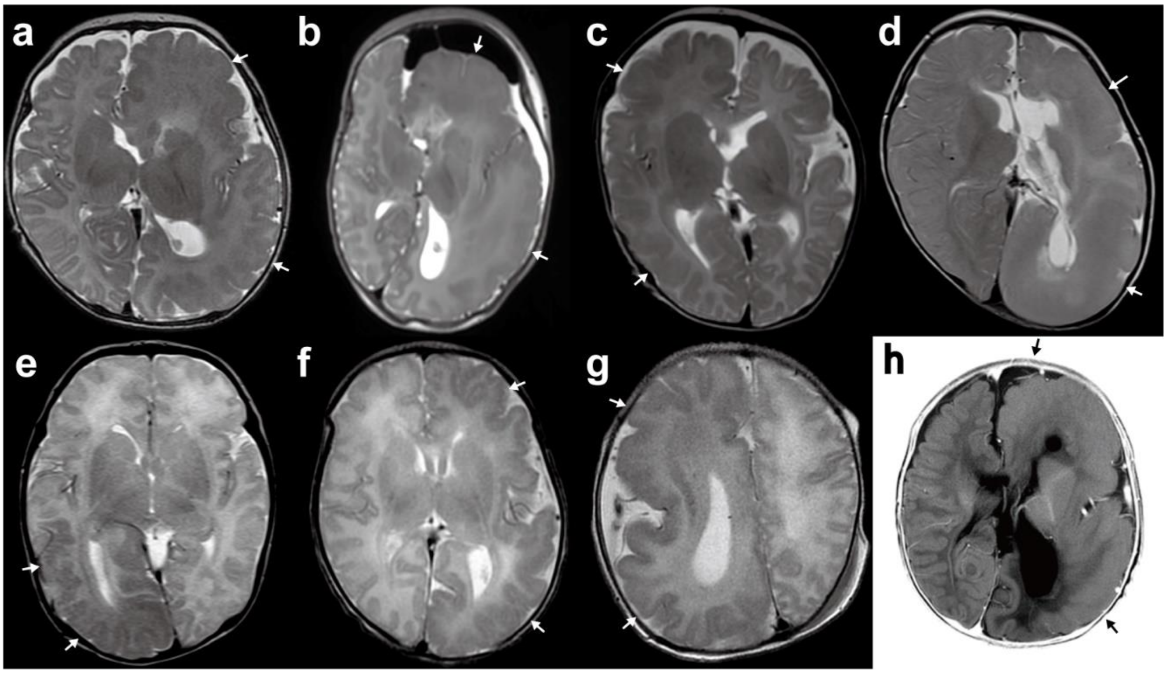

Fig. S2 Brain MRI studies of patients with hemimegalencephaly harboring somatic variants (a) Patient M28 at the age of 1 month, **(b)** patient M29 at 2 months, **(c)** patient M30 at 2 months, **(d)** patient M44 at 14 months, **(e)** patient M53 at 2 days, **(f)** patient M54 at 11 days, **(g)** patient M55 at 1 day, and **(h)** patient M56 at 3 months. T2-weighted brain MRI (a-g) and T2-reversed MRI (h) are shown. Brain MRI showing hemispherical irregular gyri (arrows) with blurred junctions between the cortex and white matter (a, b, c, d, g, and h). The patients showed hemispherical hypertrophy compatible with hemimegalencephaly.

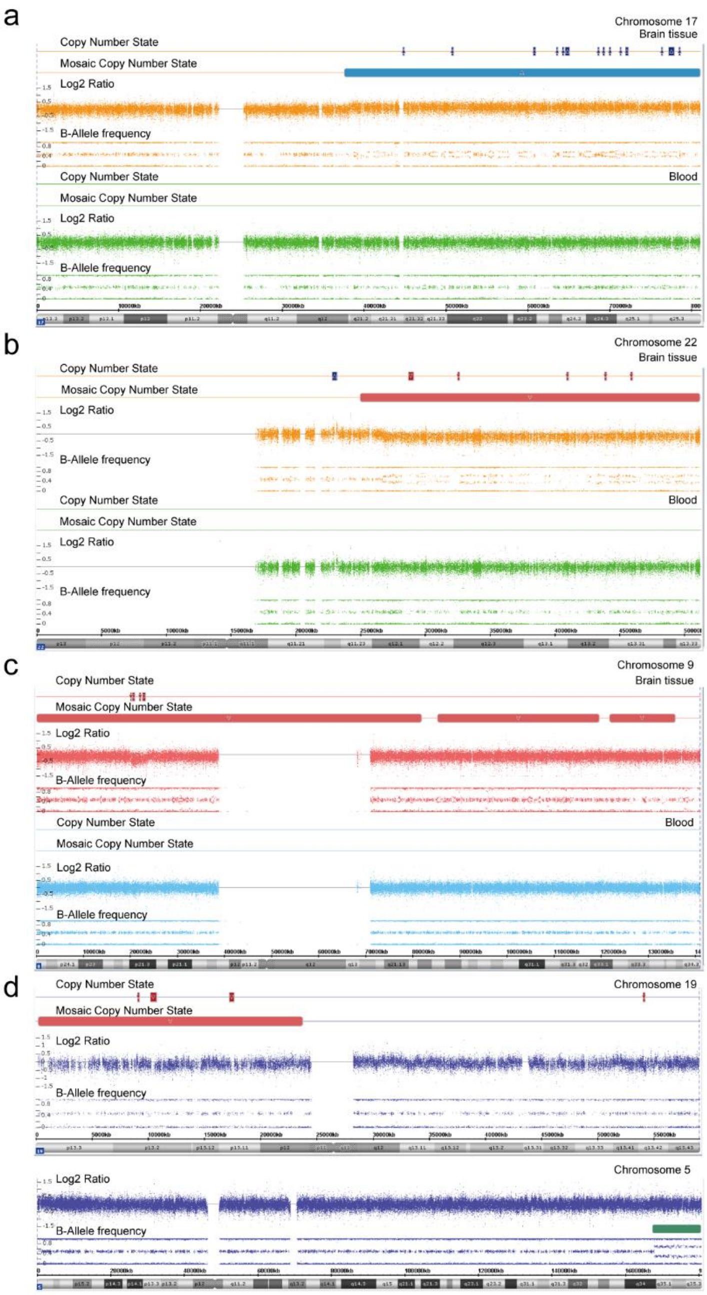

Fig. S3 Results of the SNP array in three patients in this study (a) Somatic duplication at 17q12-q25.3 and **(b)** somatic deletion at 22q11.23-q13.33 only in the brain of patient F43. Yellow and green plots show data from the brain and blood, respectively. **(c)** Somatic deletion of the entire chromosome 9 in the brain of patient F70. Pink and light blue plots show the data from the brain and blood, respectively. **(d)** Somatic deletion at 19p13.3-p12 (upper panel) and somatic copy-neutral loss of heterozygosity at 5q34-q35.3 (lower panel) in the brain of patient F30. Horizontal bars show a mosaic loss in red, mosaic gain in blue, and mosaic copy-neutral loss of heterozygosity in green.

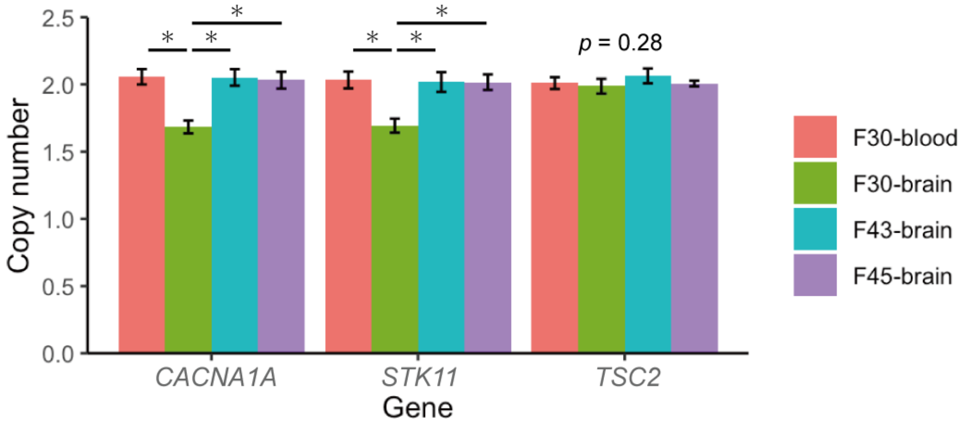

Fig. S4 Confirmation of somatic deletion of chromosome 19 in affected brain tissue from patient F30 using ddPCR *CACNA1A* at 19p13.13 and *STK11* at 19p13.3 were mapped within the somatic deletion observed using the SNP array. *TSC2* at 16p13.3 was used as a non-deleted control gene. *CACNA1A* and *STK11* are associated with epilepsy and hamartoma, respectively. Three technical replicate measurements were repeated three times for *CACNA1A* and *STK11,* and one time for *TSC2.* The data were normalized to that of the F45 brain. Statistical evaluation was performed using one-way ANOVA followed by Dunnett's post-hoc test. Data presented as mean ± SD; *, $P < 0.0001$.

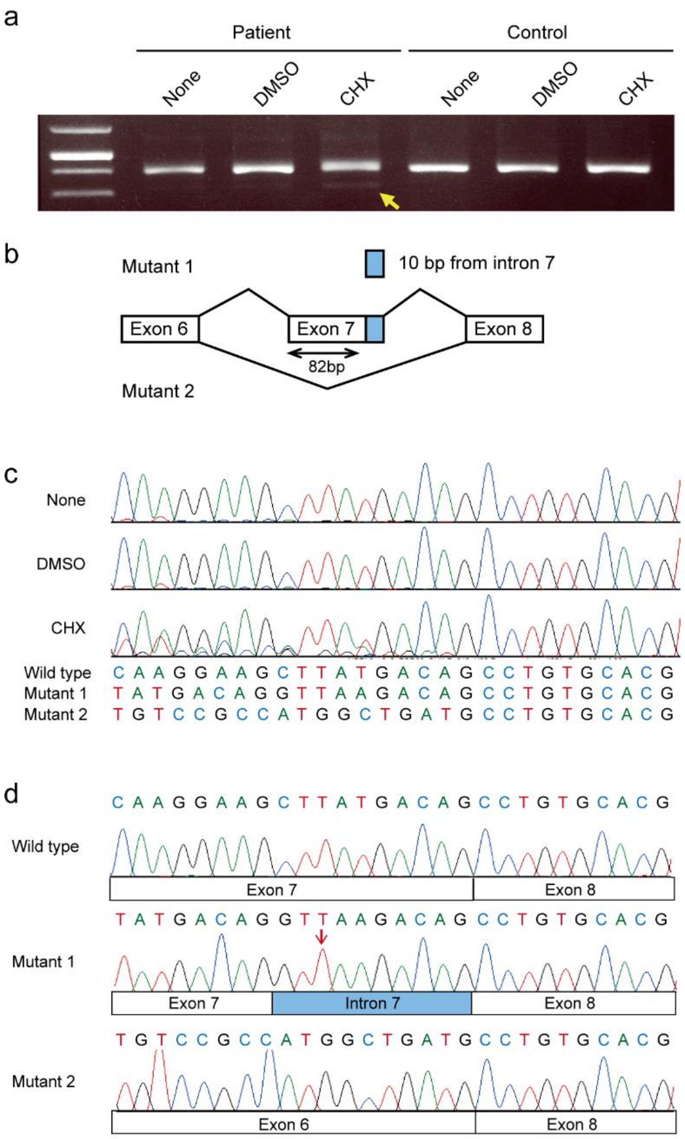

Fig. S5 cDNA analysis of a somatic splicing variant of *NPRL3* in lymphoblastoid cells from patient F38 The splice variant c.629+2_3insT of *NPRL3* showed skipping exon 7 or retention of 10 bp of intron 7. Cycloheximide (CHX) treatment determined the nonsense-mediated mRNA decay of both mutant products. **(a)** Agarose gel electrophoresis of reverse transcriptase (RT)-PCR products. The arrow shows an exon 7 skipping band (82 bp deletion). **(b)** Schematic representation of the splicing change caused by c.629+2_3insT, resulting in NM_001077350: r.[548_629del, 629_630ins[629+1_629+2;u:629+3_629+9]]. **(c)** Sanger sequencing of RT-PCR products. **(d)** Sanger sequencing of TA-cloning from RT-PCR products of a CHX-treated sample. The red arrow in Mutant 1 shows the variant position of c.629+2_3insT. DMSO, Dimethyl sulfoxide.

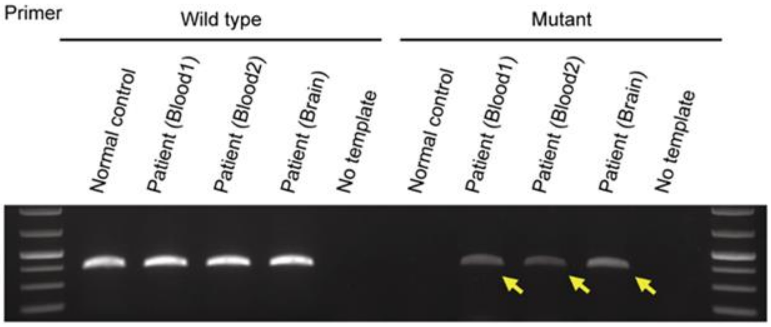

Fig. S6 Allele-specific PCR for *TSC2* somatic variant in the peripheral blood leukocytes from patient F68 Deep sequencing identified c.1418_1422del (p.Leu473Hisfs*7) of *TSC2* with a VAF of 3.6% in brain tissue, while the allele frequency was 0.03–0.4% in peripheral blood leukocytes. Allele-specific PCR confirmed the very-low-frequency variant in peripheral blood leukocytes and the brain (arrows). Blood 2 was re-extracted from stored frozen Blood 1.

Table S1 Pathological types and genetic analyses of the 64 patients enrolled in this study

ID	Pathological type	DNA: Blood/Saliva	DNA: Brain	TS: Haloplex	TS: xGen	WES: Blood/Saliva	WES: Brain	Microarray	Aberrant gene
F02[a]	FCD type IIB	+Saliva	+	-	+	+	+	-	
F04[a]	FCD type IIB	+	+	-	-	+	+	-	*TUBB3* [e]
F06[a]	FCD type IIB	+	+	+	+	+	+	+	
F08[a]	FCD type IIB	+	+	+	-	+	+	+	*MTOR*
F09[a]	FCD type IIB	+	+	+	+	+	+	+	
F11[a]	FCD type IIA	+Saliva	+	+	+	+	+	+	
F12[a]	FCD type IIA	+	+	+	+	+	+	+	
F13[a]	FCD type IIA	+	+	+	+	+	+	+	
F14[a]	FCD type IIA	+Saliva	+	+	+	+	+	+	
F15[a]	FCD type IIA	+Saliva	+	+	+	+	+	+	
F16[a]	FCD type IIB	+	+	-	+	+	+	-	
F19[a]	FCD type IIB	+	+	+	+	+	+	+	
F20[a]	FCD type I	+Saliva	+	+	+	+	+	+	
F21[a]	FCD type I	+Saliva	+	+	+	+	+	-	
F22[a]	FCD type I	+	+	+	+	+	+	+	
F23[a]	FCD type I	+	+	+	+	+	+	+	
F24[a]	FCD type I	+Saliva	+	+	+	+	+	+	
F25[a]	FCD type I	+	+	+	+	+	+	+	
F26	FCD type IB	+	+FFPE	-	-	+	+	-	*AKT3*
F29	MCD	+	+FFPE	-	-	-	+	-	*MTOR*
F30	Hippocampal sclerosis	+	+	+	+	+	+	+	chr19p del
F31	Astrocytoma grade 1	+	+	-	+	+	+	+	
F32	FCD type IB	+	+	+	-	-	-	-	*BRAF*
F33	FCD type IIB	+	+	+	+	+	+	+	
F34	Polymicrogyria	+	+	-	+	+	+	+	
F36	FCD type IIB	+	+	+	-	-	-	-	*TSC2*
F38	FCD type IIB	+	+	+	-	-	-	-	*NPRL3*
F39	FCD type IIIb (Ganglioglioma and FCD type IA)	+	+	+	-	-	-	-	*BRAF*
F40	FCD type IIB	+	+	+	+	+	+	+	*MTOR*
F41	FCD type IIB	+	+	+	+	+	+	+	*TSC2*
F43	FCD type IIB	+	+	+	+	+	+	+	*DEPDC5* and SV
F44	FCD type IIB	+	+	+	-	-	-	-	*TSC2*
F45	FCD type I	+	+	+	-	+	+	-	*SLC35A2*
F46	FCD type IIB	+	+	+	-	-	-	-	*MTOR*
F48	FCD type IB	+	+	+	+	-	-	-	*MAP2K1*
F49	FCD type IIB	+	+	+	-	-	-	-	*TSC2*
F50	FCD type IIB	+	+	+	-	-	-	-	*MTOR*
F53	FCD type IIA	+	+	+	-	-	-	-	*DEPDC5* [e]
F54	FCD type IIB	+	+	+	-	-	-	-	*MTOR*
F55	FCD type IIA	+	+	+	+	+	+	+	
F56	FCD type IIA	+	+	+	+	+	+	-	
F57	FCD type IIB	+	+	+	-	-	-	-	*MTOR*
F61	FCD type IIB	+	+	+	+	-	-	-	*TSC2* [f]
F62	FCD type IIA	+	+	+	+	+	+	-	
F63	No remarkable change[c] (insufficient sample volume)	+	+	+	-	-	-	-	*NPRL3* [e]
F64[b]	FCD type IIB	-	+	+	-	-	-	-	*MTOR*
F65	No remarkable change[c] (insufficient sample volume)	+	+	+	+	+	+	-	
F66	Pilocytic astrocytoma	+	+	+	+	+	+	-	
F67	FCD type I	+	+	+	-	+	+	-	*PTPN11*
F68	FCD type IIB, TSC	+	+	+	-	-	-	-	*TSC2*
F69	Microscopic MCD	+	+	+	+	+	+	-	
F70	Ganglioglioma	+	+	+	+	+	+	+	*TSC1* (chr9 del)
F71	FCD type IIB	+	+	+	+	+	+	-	
F72	FCD type IIA	-	+FFPE	-	+	-	-	-	*PIK3CA*
M28	No remarkable change (insufficient sample volume)[d]	+	+	+	-	-	-	-	*PIK3CA*
M29	HME	+	+	+	-	-	-	-	*PIK3CA*
M30	Atypical neuron, compatible with HME	+	+	+	-	-	-	-	*MTOR*
M44	-[d]	-	+	+	-	-	-	-	*MTOR*
M50	Microscopic MCD[d]	+	+	+	-	-	-	-	
M52	-[d]	-	+	+	-	-	-	-	*PIK3CA*
M53	HME	+	+	-	+	-	-	-	*PIK3CA*
M54	No remarkable change[d]	+	+	+	+	-	-	-	*AKT3*
M55	HME	+	+	-	+	-	-	-	*PIK3CA*
M56	Polymicrogyria, nodular heterotopia[d]	+	+	-	-	-	-	-	*PIK3CA*
	N	60	64	53	37	36	37	23	37

+ = available or performed; - = unavailable or not tested; FFPE = formalin-fixed paraffin-embedded; MCD = malformation of cortical development; TS = targeted sequencing; WES = whole-exome sequencing

[a] Previously reported patients without any candidate variants by Nakashima *et al* (reference 3). [b] previously reported by Kato *et al* (reference 2). [c] clinically FCD. [d] clinically HME, [e] germline variant, [f] germline and somatic variants.

Table S2 Clinical summary and genetic variants in patients with FCD or other pathological types in this study

ID	Pathological type	Sex	Age at onset	Seizure type	Age at Brain MRI	Location of lesion	Age at surgery	Germline/ Somatic	Gene	Variant	Method		VAF (%) in validation			Reported/ Novel variant
											Detection	Validation	Control	Blood	Brain	
F53	FCD type IIA	M	2d	FBTCS, daily	20d	Rt temporoparietooccipital	3m	Germline	*DEPDC5*	c.982C>T p.(Arg328*)	TS	Sanger	NA	NA	NA	Reported
F72	FCD type IIA	F	1y2m	FBTCS, monthly; focal motor, hourly	1y3m	Lt frontal	2y	Somatic	*PIK3CA*	c.1035T>A p.(Asn345Lys)	TS	DS	0.1	NA	15 (proximal from seizure focus), 16.8 (intermediate), 12.8 (distal)	Reported
F04	FCD type IIB	M	6y	FAS daily, FIAS FBTCS monthly	26y8m	Rt Parietal	26y9m	Germline	*TUBB3* [b]	c.520A>G p.(Lys174Glu)	WES	Sanger	NA	NA	NA	Novel
F08	FCD type IIB	M	2m	FIAS daily	3y	Lt parietal	3y	Somatic	*MTOR*	c.4339_4353del p.(Ala1447_Glu1451del)	WES	DS	0	0	1.8	Novel
F36	FCD type IIB	M	12m	Motion arrest, daily	1y4m	Rt temporooccipital	1y5m	Somatic	*TSC2*	c.2492C>T p.(Thr831Met)	TS	DS	0.1	0.1[c]	1.4	Novel
F38	FCD type IIB	F	8m	Focal motor seizure with ocular deviation to the right, FBTCS, weekly	2y	Lt orbitofrontal	3y	Somatic	*NPRL3*	c.629+2_3insT	TS	DS	0	32.5	32.9	Novel
F40	FCD type IIB	F	4y	Motion arrest and staring, daily	13y	Rt frontal	14y	Somatic	*MTOR*	c.4447T>C p.(Cys1483Arg)	TS	DS	0	0	0.6	Reported
F41	FCD type IIB	M	6y	FBTCS, monthly	13y	Lt parietal	13y	Somatic	*TSC2*	c.5227C>T p.(Arg1743Trp)	TS	DS	0	0	1	Novel
F43	FCD type IIB	M	5y	Status epilepticus of FBTCS	NA	Rt frontal	6y	Somatic	*SV*	22q11.23q13.33del (DEPDC5) and 17q12q25.3dup	WES	CMA	0 (2 copies)	0 (2 copies)	20-25 (1.5 copies and 2.4 copies)	Novel
								Somatic	*DEPDC5*	c.856C>T p.(Arg286*)	TS	DS	0	0.1[c]	1.8	Reported
F44	FCD type IIB	M	0m	Focal motor at Rt face at 0m, FBTCS at 3y, weekly	6y	Rt frontal	6y	Somatic	*TSC2*	c.1513C>T p.(Arg505*)	TS	DS	0	0	1.6	Novel
F46	FCD type IIB	F	14y	Focal motor at Rt face, weekly	39y	Lt frontal	39y	Somatic	*MTOR*	c.4376C>A p.(Ala1459Asp)	TS	ddPCR	0.007	0	2.56	Reported
F49	FCD type IIB	F	3y	FIAS, monthly to weekly	4y	Lt parietooccipital	5y	Somatic	*TSC2*	c.5228G>A p.(Arg1743Gln)	TS	DS	0.1	0.1[c]	1.3	Reported
F50	FCD type IIB	M	6y	FIAS, daily	25y	Lt parietal	25y	Somatic	*MTOR*	c.5930C>A p.(Thr1977Lys)	TS	DS	0	0	2.6	Reported
F54	FCD type IIB	F	13y	FIAS, daily	53y	Rt frontal	53y	Somatic	*MTOR*	c.4379T>C p.(Leu1460Pro)	TS	ddPCR	0	0	1.84	Reported
F57	FCD type IIB	M	6y	FBTCS, focal motor, weekly	15y	Lt parietal	15y	Somatic	*MTOR*	c.6644C>A p.(Ser2215Tyr)	TS	ddPCR	0	0	3.18	Reported
F61	FCD type IIB	F	1y	FIAS, weekly	2y	Rt frontotemporal	2y	Somatic	*TSC2*	c.4375C>T p.(Arg1459*)	TS	DS	0	0	0.6	Novel
								Germline	*TSC2*	c.4960G>A p.(Gly1654Ser)	TS	Sanger	NA	NA	NA	Novel
F64	FCD type IIB	F	53y	FIAS weekly	56y	Rt parietal & frontal	57y	Somatic	*MTOR*	c.4448G>A p.(Cys1483Tyr)	TS	DS	0	NA	4	Reported
F68	FCD type IIB, TSC	F	2m	Epileptic spasms, hourly	9m	Lt occipital	9m	Somatic	*TSC2*	c.1418_1422del p.(Leu473Hisfs*7)	TS	DS/ ASP	0	0.03-0.4	3.6	Novel
F26	FCD type IB	F	0m	Focal motor, daily	1y	Bilateral, Rt side dominant	1y3m	Somatic	*AKT3*	c.49G>A p.(Glu17Lys)	WES	DS	0	0	4.9	Reported
F32	FCD type IB	M	2y	Staring, daily	12y	Lt parahippocampal	13y	Somatic	*BRAF*	c.1799T>A p.(Val600Glu)	TS	DS	1	0.3[c]	7.5	Reported
F45	FCD type I	F	4m	Epileptic spasms, hourly	1y11m	Rt frontal	1y11m	Somatic	*SLC35A2*	c.844G>A p.(Gly282Arg)	WES	DS	0.1	0.1[c]	5.4	Novel
F48	FCD type IB	M	4y	FIAS, FBTCS, monthly	23y	Rt hippocampus	23y	Somatic	*MAP2K1*	c.173_187del p.(Gln58_Glu62del)	TS	DS	0	0	0.8	Novel
F67	FCD type I	F	11y	FIAS, weekly	18y (normal)	Rt frontal	20y	Somatic	*PTPN11*	c.178G>C p.(Gly60Arg)	WES	DS	0.1	0.1[c]	2.3	Novel
F29	MCD	M	3d	Focal motor, hourly	3d	Bilateral, Rt side dominant	2m	Somatic	*MTOR*	c.4379T>C p.(Leu1460Pro)	WES	ddPCR	0	0	2.3	Reported
F39	FCD type IIIb (Ganglioglioma and FCD type IA)	M	3m	Focal motor, daily	5m	Rt temporal	5m	Somatic	*BRAF*	c.1799T>A p.(Val600Glu)	TS	DS	0	0	0 (temporal lobe), 29.1(amygdala)	Reported
F70	Ganglioglioma	M	18y	FBTCS, monthly	21y	Lt temporal	21y	Somatic	SV	chr 9 del (*TSC1*)	WES	CMA	0 (2 copies)	0 (2copies)	15-20 (1.6-1.7 copies)	Reported
F30	Hippocampal sclerosis	M	6m	Focal motor with eye deviation to the right, daily	2y7m	Rt hippocampus	4y	Somatic	SV	19p13.3p12 del	CMA	ddPCR	0 (2 copies)	0 (2 copies)	20 (1.7 copies)	Novel
F63	No remarkable change (insufficient sample volume)[a]	M	2m	FIAS	1y4m	Lt parietal	1y11m	Germline	*NPRL3*	c.1270C>T p.(Arg424*)	TS	Sanger	NA	NA	NA	Reported

ASP = allele specific PCR; CMA = chromosomal microarray; ddPCR = droplet digital PCR; DS = amplicon deep sequencing; FAS = Focal aware seizure; FBTCS = focal to bilateral tonic-clonic seizure; FIAS = focal impaired awareness seizure; Lt = left; NA =not available; Rt = right; TS = targeted sequencing; VAF = variant allele frequency; WES = whole-exome sequencing.

[a]clinically suspected FCD. [b]*TUBB3* abnormality causes cortical dysplasia, complex, with other brain malformations 1 with autosomal dominant inheritance (OMIM# 614039). [C] These data were considered false positive due to similar error rates in the short-read sequencer (0.1%).[1]

Refseq accession number: *AKT3* (NM_005465), *BRAF* (NM_004333), *DEPDC5* (NM_001242896), *MAP2K1* (NM_002755), *MTOR* (NM_004958), *NPRL3* (NM_001077350), *PIK3CA* (NM_006218), *PTPN11* (NM_002834), *SLC35A2* (NM_001042498), *TSC2* (NM_000548), and *TUBB3* (NM_006086).

Table S3 Clinical summary and genetic variants in patients with hemimegalencephaly in this study

ID	Pathological type	Sex	Age at onset	Seizure type	Age at Brain MRI	Location of lesion	Age at surgery	Germline/ Somatic	Gene	Variant	Method		VAF(%)			Reported/ Novel variant
											Detection	Validation	Control	Blood	Brain	
M28	No remarkable change (insufficient sample volume)[a]	F	1m	Epileptic spasms, hourly	1m	Lt hemisphere	4m	Somatic	*PIK3CA*	c.1624G>A p.(Glu542Lys)	TS	DS	0	0.2[b]	34.9 (gyrus), 21.7 (heterotopia)	Reported
M29	HME	M	1d	Tonic seizures, hourly	2m	Lt hemisphere	2m	Somatic	*PIK3CA*	c.1624G>A p.(Glu542Lys)	TS	DS	0	0	27.2	Reported
M30	atypical neuron, compatible with HME	M	20d	FBTCS, weekly	2m	Rt hemisphere	5m	Somatic	*MTOR*	c.6644C>T p.(Ser2215Phe)	TS	ddPCR	0.025	0.016[b]	16.28	Reported
M44	-[a]	M	0d	Tonic or clonic seizures in cluster	1y2m	Lt hemisphere	1y2m	Somatic	*MTOR*	c.4348T>G p.(Tyr1450Asp)	TS	DS	0	NA	6.2, 4.5, 4.2 (tumor like), 5.0 (HME cortex)	Reported
M52	-[a]	F	20d	FBTCS, hourly	NA	Lt hemisphere	9m	Somatic	*PIK3CA*	c.1633G>A p.(Glu545Lys)	TS	DS	0	NA	11.3	Reported
M53	HME	F	1d	Epileptic spasms, daily	2d	Rt hemisphere	4m	Somatic	*PIK3CA*	c.3140A>G p.(His1047Arg)	TS	DS	0	0	16.8 (pars opercularis), 22.3 (temporal lobe), 15.2 (lipoma)	Reported
M54	No remarkable change[a]	F	2d	Focal motor, FBTCS, daily	11d	Lt hemisphere	25d	Somatic	*AKT3*	c.49G>A p.(Glu17Lys)	TS	DS	0	0	1.1	Reported
M55	HME	F	16d	focal motor, epileptic spasms, daily	1d	Rt hemisphere	5m	Somatic	*PIK3CA*	c.1624G>A p.(Glu542Lys)	TS	DS	0	0	25.4	Reported
M56	Polymicrogyria, nodular heterotopia[a]	M	0m	Focal motor with eye deviation to the left daily, FBTCS, daily	3m	Lt hemisphere	4m	Somatic	*PIK3CA*	c.1633G>A p.(Glu545Lys)	Sanger	DS	0	0	14.6, 11.9 (temporal lobe), 8.3 (Hippocampus), 7.1 (Amygdala), 14.3 (skin)	Reported

ddPCR = droplet digital PCR; DS = amplicon deep sequencing; FBTCS = focal to bilateral tonic-clonic seizure; FIAS = focal impaired awareness seizure; GTC = generalized tonic-clonic seizure; Lt = left; Rt = right; TS = targeted sequencing; VAF = variant allele frequency.
[a]Clinically HME, [b] These data were considered false positive because VAFs in the blood were smaller in controls as per ddPCR or similar to the error rate for the short-read sequencer (0.1%).[1]
Refseq accession number: *AKT3* (NM_005465), *MTOR* (NM_004958), *PIK3CA* (NM_006218).

Table S4 In-silico predictions of novel missense and in-frame variants identified in this study

ID	Germline/ Somatic	Gene	Variant	SIFT		Polyphen2 HumVar		CADD	PROVEAN	
F04	Germline	*TUBB3*	NM_006086: c.520A>G p.(Lys174Glu)	0.01	Damaging	0.981	Probably Damaging	25	NA	
F08	Somatic	*MTOR*	NM_004958: c.4339_4353del p.(Ala1447_Glu1451del)	NA		NA		NA	-31.56	Deleterious
F36	Somatic	*TSC2*	NM_000548: c.2492C>T p.(Thr831Met)	0.01	Damaging	0.998	Probably Damaging	26.9	NA	
F41	Somatic	*TSC2*	NM_000548: c.5227C>T p.(Arg1743Trp)	0.00	Damaging	0.999	Probably Damaging	25.6	NA	
F45	Somatic	*SLC35A2*	NM_001042498: c.844G>A p.(Gly282Arg)	0.03	Damaging	1.000	Probably Damaging	24.3	NA	
F48	Somatic	*MAP2K1*	NM_002755: c.173_187del p.(Gln58_Glu62del)	NA		NA		NA	-26.14	Deleterious
F61	Germline	*TSC2*	NM_000548: c.4960G>A p.(Gly1654Ser)	0.01	Damaging	0.998	Probably Damaging	23.3	NA	
F67	Somatic	*PTPN11*	NM_002834: c.178G>C p.(Gly60Arg)	0.00	Damaging	0.997	Probably Damaging	29.1	NA	

SIFT, https://sift.bii.a-star.edu.sg/; PolyPhen-2, http://genetics.bwh.harvard.edu/pph2/; CADD, https://cadd.gs.washington.edu/; PROVEAN, https://www.jcvi.org/research/provean

NA = not available

Table S5 Somatic structural variations identified in this study

ID	Whole-exome sequencing	SNP array			
	Structural variations	Structural variations	CN state	Size (Mb)	Protein coding genes
F30	Not detected	arr[GRCh37] 5q34q35.3(167826913-180719788)x2 mos hmz, 19p13.3p12(260911-23765479)x1-2	2, 1.66[a]	12.9, 23.5	128, 596
F43	seq[GRCh37]dup(17)(q21.1q25.3), del(22)(q12.1q13.33) NC_000017.10:g.38140461-80963293dup NC_000022.10:g.26936736-51143587del	arr[GRCh37] 17q12q25.3(37,623,726-81,041,938)x2-3, 22q11.23q13.33(24,995,260-51,197,838)x1-2	2.39, 1.53	43.4, 26.2	643, 310 (*DEPDC5*)
F70	seq[GRCh37]del(9)(p24.1q34.3) NC_000009.11:g.8465365-140347371del	arr[GRCh37] 9p24.3q21.31(203861_135775224)x1-2	1.58-1.72	135	629 (*TSC1*)

a 1.7 copy in ddPCR

SNP = single nucleotide polymorphism; CN = Copy number

Supplementary references

1 Fox EJ, Reid-Bayliss KS, Emond MJ, Loeb LA (2014) Accuracy of Next Generation Sequencing Platforms. Next Gener Seq Appl 1: Doi 10.4172/jngsa.1000106

2 Kato M, Kada A, Shiraishi H, Tohyama J, Nakagawa E, Takahashi Y, Akiyama T, Kakita A, Miyake N, Fujita Aet al (2022) Sirolimus for epileptic seizures associated with focal cortical dysplasia type II. Ann Clin Transl Neurol 9: 181-192 Doi 10.1002/acn3.51505

3 Nakashima M, Saitsu H, Takei N, Tohyama J, Kato M, Kitaura H, Shiina M, Shirozu H, Masuda H, Watanabe Ket al (2015) Somatic Mutations in the MTOR gene cause focal cortical dysplasia type IIb. Ann Neurol 78: 375-386 Doi 10.1002/ana.24444
